# Snus use in football: the threat of a new addiction?

**DOI:** 10.5114/biolsport.2024.130050

**Published:** 2023-07-24

**Authors:** Daniel Read, Sarah Carter, Phil Hopley, Karim Chamari, Lee Taylor

**Affiliations:** 1 Institute for Sport Business, Loughborough University London, London, United Kingdom; 2 Faculty of Health, Exercise and Sports Science, Charles Darwin University, Darwin, NT 0810, Australia; 3 Cognacity Wellbeing, London, United Kingdom; 4 Aspetar Sports Medicine and Orthopedic Hospital, FIFA Medical Centre of Excellence, Doha, Qatar; 5 School of Sport, Exercise and Health Sciences, Loughborough University. National Centre for Sport and Exercise Medicine (NCSEM), Loughborough, United Kingdom; 6 Sport and Exercise Discipline Group, Faculty of Health, University of Technology Sydney, Moore Park, NSW, Australia; 7 Human Performance Research Centre, University of Technology Sydney (UTS), Sydney, Australia

**Keywords:** Nicotine, Substance use, Performance, Mental health, Dependence, Football medicine

## Abstract

The use of Snus, an oral nicotine pouch, is becoming increasingly common in English professional football. As a nicotine product, Snus raises important questions about health and performance for practitioners. The purpose of this short communication is to explain the current regulatory status of Snus, performance relatedeffects, and associated health outcomes. Further, based on player statements and evidence from the general public, we argue that Snus is used as a coping mechanism to deal with the stressors of professional football. Accordingly, the communication concludes with guidance for club-level multidisciplinary interventions to support player welfare, aimed at reducing Snus use as well as future research recommendations.

## INTRODUCTION

High performance expectations, employment insecurity, fatigue, injury, frequent travel, and intense media scrutiny are hallmark stressors of the professional men’s football environment conducive to mental health issues [1]. Equally, strong socio-cultural and experiential barriers exist in football that often prevent players from seeking psychological help [2]. Accordingly, the prevalence of stress and mental health problems in male professional footballers (e.g., anxiety, distress, depression) are considerable, mirroring rates in the general population including mild to severe presentation [1]. Athletes may turn to a range of mood-altering substances such as alcohol, cigarettes, cannabis, and/or painkillers used recreationally to cope with the mental load/stress of a sporting career [3]. Mood-altering substances often negatively influence well-being [4], for example, cannabis use is becoming recognised as a modifiable risk factor for several adverse effects on human health, including mental illness [5].

Snus use is common in professional male football players, attracting negative attention from practitioners, clinicians and the popular press [6–10]. It is an oral, smokeless tobacco product containing nicotine that is placed between the gum and upper lip. Player testimony alongside journalistic investigations confirm Snus is predominantly used recreationally to relax [8, 10–12] and like smoking, is a substance commonly used to regulate mood [13]. However, whether Snus negatively affects player health or performance is not well established.

We provide a summary of the health and performance effects of Snus and nicotine, arguing that Snus use should not be considered an independent behaviour. Rather, a response to significant football-specific occupational stress warranting an interdisciplinary approach to reduce its widespread use, yet poorly understood effects, on player health and performance.

### Is Snus legal?

Snus is not prohibited by the World Anti-Doping Agency (WADA) as a performance enhancing substance or controlled substance. Nicotine is however, on the WADA 2023 Monitoring Programme for in-competition use as a stimulant. Nicotine primarily acts as a neuroregulatory agent on neuronal nicotinic acetylcholine receptors in the central and peripheral nervous system, releasing dopamine via the mesolimbic pathway [14]. It has stimulatory effects in lower doses whilst depressing the central nervous system in higher doses leading to feelings of relaxation [15]. Analysis of 60,802 in-competition antidoping urine samples taken in Italy from 2012–2020 reported that on average, 22.7% (15.2–32.5%) of all samples indicated nicotine intake, increasing to 29% (18.4–40.4%) in football [16]. The nicotine content in commercially available pouched Snus from Europe is comparable to a cigarette (approx. 15mg per product) [17]. However, it leads to significantly higher plasma nicotine concentration than smoking due to the longer use duration [17]. The nicotine content and carcinogen content of Snus products varies considerably between products [18]. Indeed, Swedish Snus has greater levels of unionized nicotine in comparison to US products meaning nicotine can be absorbed quicker across mucous membranes leading to a ‘greater nicotine reward’ [19].

It is not illegal to possess or consume Snus in the UK, but it is illegal to sell. Player testimonies suggest that purchasing Snus online through social media and illegal websites or from other players is common. This raises questions about substance propriety (e.g., contaminated products that may trigger anti-doping rule violations) alongside legal and employment implications for any players caught selling Snus to teammates [7]. Ultimately, whilst nicotine remains legal from an anti-doping perspective, there is no policy incentive for players to change their behaviour. Instead, Snus use should be treated as a matter of professionalism, like alcohol and smoking, with concern concentrated on player health and team performance.

### Does Snus Impact Performance or Recovery?

A recent review of the limited studies concerning oral nicotine and Snus use in sport concluded that performance enhancement was unlikely and performance may even diminish [6]. A broader review of nicotine’s impact on performance (aerobic, anaerobic and muscular) also concluded that ergogenic effects were unlikely, however, the available evidence quality for this conclusion was low [20]. Opposingly, meta-analytical findings support that nicotine can enhance some cognitive abilities on tasks involving fine motor skills, attention, and memory [21]. Further, the calming effects of high-dose nicotine described by users may offer short-term protection against the impact of stress on performance. Although, determining the impact of nicotine on performance is complex and often confounded by sampling of nicotine in naïve and chronic user participants. Indirectly, nicotine use is associated with sleep impairment [22] and the deleterious effects of limited sleep on athletic performance and recovery are well documented [23]. Equally, nicotine can lead to increased metabolic energy expenditure, reduced body weight, and appetite suppression [24] meaning Snus use may challenge optimal nutritional support for athletic performance and recovery. Therefore, chronic Snus use has the potential to undermine performance/recovery via impaired sleep and potentially diet.

### Does Snus Impact Health?

**FIG. 1:**
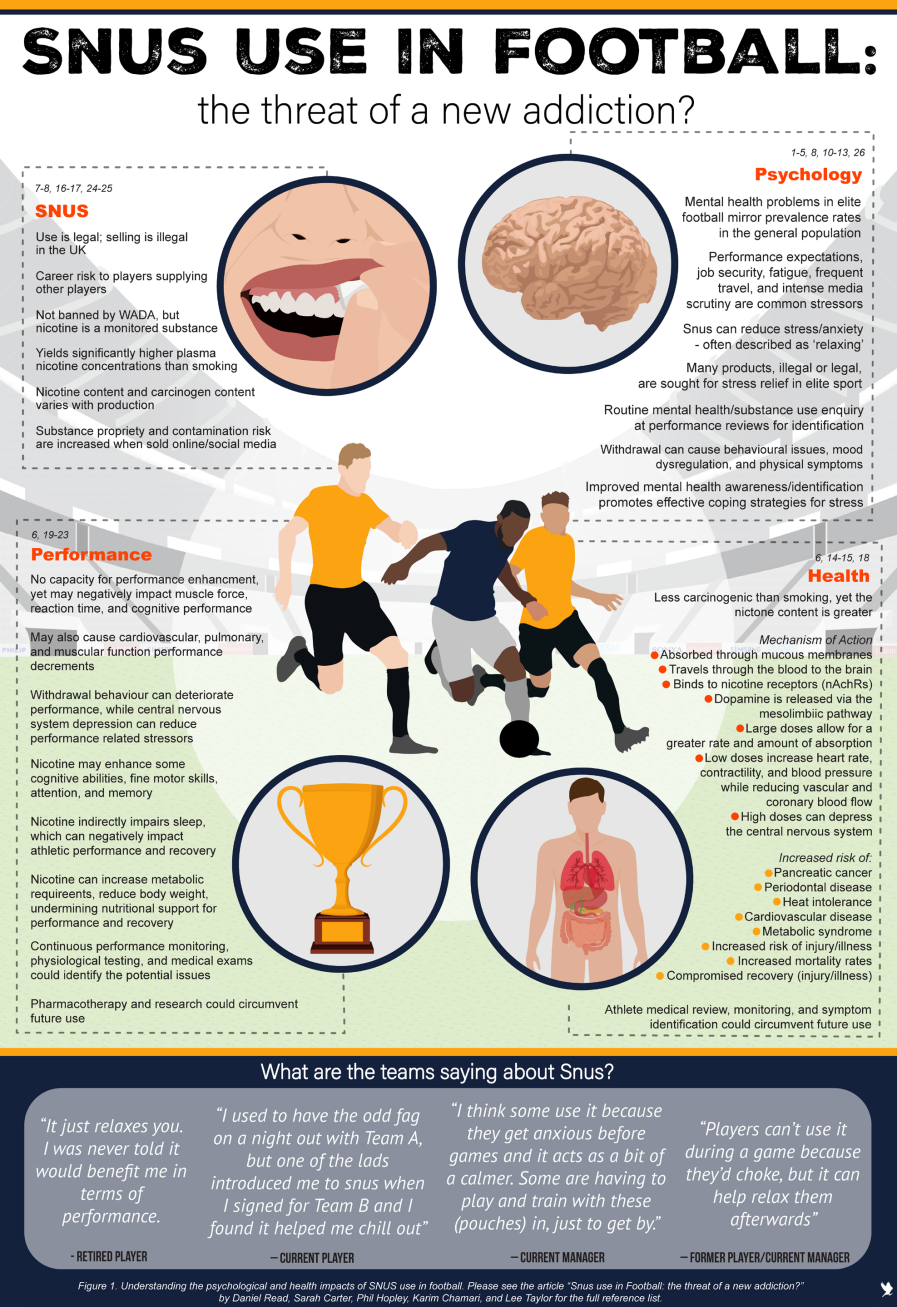
Understanding the psychological and health impacts of Snus use in football. Please see commentary for the full reference list.

Snus use has been associated with noteworthy short and long-term physiological health risks [6] linked to nicotine consumption (see Figure 1) including: (i) increased risk of periodontal disease; (ii) heat intolerance; (iii) impaired cardiovascular function; (iv) metabolic syndrome; and (v) increased mortality rates, caveated with the need for more robust studies in sport-specific populations. More broadly, meta-analytical results from military training studies indicate an association between smoking and increased injury risk [25] but there is little evidence to comment on any relationship between smokeless tobacco products, like Snus, and injury rates in high-performance athletic populations. Nicotine is a highly addictive compound and anecdotal reports suggest that dependence is becoming more common in football [8]. Like other addictions, nicotine dependence is associated with mental health issues and may lead to adverse physical and psychological withdrawal symptoms [26]. For example, short-term abstinence from nicotine can lead to intensified moodrelated symptoms including anxiety and depression [27]. On balance, using Snus as a stress-coping method has the potential to cause social, physical, and mental harm undermining players’ performance and recovery.

### Reducing Snus Use

A central premise of this manuscript is that Snus use can be viewed as a maladaptive stress management strategy for football-related occupational stress. Smoking cessation, appetite suppression, and pre-match psychological reassurance have been cited as motivations for Snus use. Yet anecdotally the most frequently stated reason is for recreational relaxation as shown in Figure 1, aligning with research examining smokeless tobacco users in sporting samples [28] and Snus use in the general public [13].

Paradoxically, Snus use may enhance the risk of poor performance, injury and ill-health, a significant stressor for professional athletes. Yet instigating changes towards more positive health behaviours in athletes remains difficult and education alone is often insufficient [29]. In practice, an interdisciplinary approach that not only provides information concerning health risks but situates Snus use within broader strategies to improve athlete mental well-being is required. For example, psychologists can help footballers to cope with underlying mental health status and sport specific stressors by understanding player history, peer-pressure, social supports, motivation for use, and providing evidence-based interventions (e.g., cognitive behavioural therapy). It is important that when discussing Snus use and potential cessation strategies that conversation is framed against factors valued by players to promote engagement, such as any potential negative impact on their performance. Likewise, routine enquiry and education by medical staff, identification of at-risk players through daily well-being reporting by staff with frequent interaction (e.g., physiotherapists, S&C staff), and monitored pharmacotherapy for nicotine dependence, could all contribute to identifying, preventing, reducing, and hopefully stopping Snus use. Lastly, given that occupational stressors are inherent in a professional football career, ongoing work to promote mental health awareness and reduce barriers to seeking mental health support may encourage more adaptive methods for coping with stress at an individual and club level [30].

## CONCLUSIONS

Recognising the inherent data limitations, the illustrative player testimonies presented in Figure 1 support a call for more research and systematic reviews examining Snus use in elite sport. We outline six future research avenues that could develop our understanding of

Snus use in football and other elite sporting populations as well as assisting sport medicine practitioners working with athletes. First, dedicated qualitative and quantitative analysis voicing the experience and motivations of Snus use in footballers that encompasses non-users to daily users should be prioritised to theorise behaviour and understand how to design behavioural interventions. Second, comprehensive prevalence surveys should be employed to accurately establish Snus use prevalence and patterns (e.g., when and how much is used). Third, as previously mentioned, the impact of nicotine use on performance is complicated warranting further studies into the impact of Snus use on physiological, cognitive, and match performance in elite samples [31]. Fourth, an objective focus on the wellbeing impacts of Snus use and dependence are essential. At present, there is scant evidence on the long-term consequences of Snus use in elite sporting populations to properly assess the behavioural risk. Epidemiological studies should therefore be undertaken to assess lifelong outcomes that include current and retired players. Fifth, staff experiences of athlete Snus use should be canvassed to ascertain field experience to guide research [32]. Finally, rigorously designed, long-term intervention studies utilising different psychological techniques can build an evidence base to support practitioners in reducing use. All of the above would inform policies and protocols for clinical decision-making aiming at supporting holistically healthy footballers.
